# A Descriptive Overview of the Medical Uses Given to *Mentha* Aromatic Herbs throughout History

**DOI:** 10.3390/biology9120484

**Published:** 2020-12-21

**Authors:** Henrique Silva

**Affiliations:** 1 Informetrics Research Group, Ton Duc Thang University, Ho Chi Minh City 758307, Vietnam; henriquesilva@tdtu.edu.vn; 2 Faculty of Pharmacy, Ton Duc Thang University, Ho Chi Minh City 758307, Vietnam

**Keywords:** *Mentha* L. genus, aromatic herb, history, pharmacognosy, therapeutics

## Abstract

**Simple Summary:**

Mints are aromatic herbs with a millenary tradition of use for diverse medicinal purposes since ancient civilizations, and they are still presently used in different clinical practices. Mints have been used since ancient Babylon, but it was in Classical Antiquity that their medical uses flourished, with major contributions from Pliny the Elder. In the Middle Ages, the increased knowledge surrounding mints came from Byzantine physicians, while, in the Modern Age, technological developments allowed the production of mint-based products, such as extracts and essential oils, which have become part of elaborate galenic formulas employed by an increasing number of physicians, and have also stimulated both scientific and artistic interests alike. In present-day medicine, several mints and mint-based products are being researched as potential therapeutic alternatives for many diseases, while also being vastly employed in food and cosmetic industries.

**Abstract:**

Mints have been among the most widely used herbs for medicinal purposes since ancient civilizations. They are still presently used for numerous purposes, including non-medicinal, which makes them economically relevant herbs. Information regarding the medical and scientific uses given to mints throughout history are vastly scattered and/or incomplete. The aim of this paper is to provide an extensive descriptive overview of the medical uses given to these herbs, highlighting both the authors in medical culture responsible for their dissemination, as well as their major galenic formulations. Databases on medical science, reference textbooks on medical history, botanics (aromatic herbs), and pharmacognosy were consulted. The use of mints remotes to Classical Antiquity, with major contributions from Pliny the Elder. In the Middle Ages, the increased knowledge surrounding mints came from Byzantine physicians, while, in the Modern Age, technological developments allowed the production of mint-based products which have become part of elaborate galenic formulas employed by an increasing number of physicians, as well as have also stimulated both scientific and artistic interests alike. In present-day medicine, several mints and mint-based products are being researched as potential therapeutic alternatives for many diseases, while also being vastly employed in food and cosmetic industries.

## 1. Introduction

Medicinal plants have been at the forefront of most medical therapies for many centuries and have been used in many societies worldwide. A large number of medical treatises have established several plant-based medicinal products as the most important components of the available therapeutic arsenal. Members of aromatic mint herbs have been among the most widely used plants throughout history for a multitude of medicinal purposes. This paper provides a thorough review of the medical uses given to mint herbs in western medicine from ancient civilizations to modern day medicine and provides recent scientific data to support the reasons behind the longevity of their use. Mint herbs consist of perennial aromatic members of the Lamiaceae family, the *Mentha* L. genus, classified into 42 species, 15 hybrids, and hundreds of subspecies, varieties and cultivars. Among the best-known mint species, we find watermint (*Mentha aquatic* L.), spearmint (*Mentha spicata* L.), wildmint (*Mentha arvensis* L.), horsemint (*Mentha longifolia* (L.) L.), pennyroyal (*Mentha pulegium* L.), and peppermint (*Mentha x piperita* L.), the last of which is a natural sterile hybrid of watermint and spearmint [1,2]. While most mints have been known to man since ancient times, it was not until 1696 that peppermint was described by English Botanist John Ray (1627–1705) in his *Synopsis Methodica Stirpium Britannicarum* [3].

Most mints invariably grow in moist environments near ponds, lakes, and rivers and require partial shade, although some species grow well in warm environments [4]. Interestingly, one of the first written references to mints, the poem *Theriaca* by Nicander of Colophon (197 BC–170 BC), Greek poet and physician, alludes to their habitat and describes them as ‘delighting in gleaming rivers’ [5]. Sterile mint hybrids, such as peppermint, only display vegetative reproduction, while the majority display sexual reproduction and contain both male and female organs [4]. It is likely that mints’ sexual dimorphism justified the comparison made between pennyroyal and the fictitious magical plant *Pantagruelion*, as featured in François Rabelais’ *Gargantua and Pantagruel* [6]. The first attempt to classify them from a botanical perspective came from Pedanius Dioscorides (c. 40–90), the renowned Greek physician and herbalist, whose teachings became a reference from classical antiquity well into the Renaissance period. A more formal classification was proposed several centuries later by Carl Nilsson Linnæus (1707–1778) in his *Species Plantarum* (The Species of Plants) published in 1753 [7], which has been further perfected in recent years based on the genetic relations between these species [8]. To contribute to the complexity of the mint taxonomy, all the different species are referred to in many medical treatises as simply mint, which prevents a more precise appreciation of the uses given to each particular species. From a pharmacognosy perspective, the therapeutic value of herbs lies in aerial parts, which can be dried and ground into powder or used fresh. If used fresh, aerial parts can be subjected to water or steam distillation to produce an essential oil that is extracted [4].

The name of the mints’ genus, *Mentha*, was probably coined by the Greek philosopher Theophrastus (371 BC–287 BC), who described several species from botanical and agricultural standpoints in his *Enquiry into Plants* [9]. The name *Mentha* probably alludes to Minthe, a figure of Greek mythology, although different ancient texts describe different versions of her myth. According to the myth told in the didactic epic *Halieutika* (On Fishing) by Oppianus of Corycus (fl. 2nd century), the Greek-Roman poet contemporary of emperor Marcus Aurelius and of Galen, Minthe was a nymph of the river Cocythus and wife of Aidoneus. But when Aidoneus raped Persephone, of the Aetnaean hill, Minthe, overtaken by jealousy, claimed to be nobler of form and more excellent in beauty than Persephone, and, for this, she was trampled upon and destroyed by Demeter, Persephone’s mother, and from the earth sprang the herb that carries her name [10]. In the versions told by Strabo (c. 64 BC–24), the Roman geographer, and by the Roman poet Ovid (43 BC–17), Minthe was beloved by Hades, and it was Persephone, the god’s jealous wife that destroyed the nymph [11,12].

The oldest written records of mint herbs are attributed to King Hammurabi of ancient Babylon (1800 BC), who prescribed them for medicinal purposes, namely gastrointestinal [13]. Mints are also referred to in the Ebers papyrus and on the walls of the temple of Horus in Edfu [14,15]. A great deal of knowledge on the mints’ usage was acquired during classical antiquity by Greek and Roman philosophers, especially Gaius Plinius Secundus (Pliny the Elder, 23–79), who described most of the medical uses that would be given to these herbs throughout the history of western medical practice in his *Naturalis Historia* (Natural History) [16], an important work that would be the reference for several posterior medical texts. Knowledge of these herbs spread throughout the Middle East, partly due to the conquests of Alexander the Great (356 BC–333 BC) and later to the Crusades [17]. Given their many uses, mints became valuable herbs and were probably used as currency by the Pharisees, as can be inferred from two passages from the Bible: ‘(…) Woe to you, scribes and Pharisees, hypocrites! For you tithe mint, dill, and cumin, and have left undone the weightier matters of the Law: justice, mercy, and faith (…)’ (Matthew 23:23) and ‘(…) But woe to you Pharisees! For you tithe mint and rue and every herb, but you bypass justice and the love of God (…)’ (Luke 11:42) [18]. Several references are also found in the Babylonian Talmud, a compilation of Jewish teachings on several knowledge areas that contains many pieces of practical advice of a medical nature [19]. In the Middle Ages, medical and scientific learning shifted to Constantinople, the capital of the Byzantine Empire, with the majority of important references to mints coming from Islamic physicians, such as Ibn Sinna (Avicenna, 980–1037), author of *Canon of Medicine* [20]. In Christian Europe, medical knowledge was kept mostly in universities and monasteries by physician-monks, where the teachings of classical philosophers were still followed [17]. Charlemagne (c. 747–814), leader of the Holy Roman Empire and founder of the first medical school in Salerno in the 9th century, ordered medicinal plants, in which mints were already included, to be cultivated inside state-owned land, as stated in his *Capitulare de villis* [21]. The Renaissance period allowed a change to medico-scientific paradigms and the progressive abandonment of empiric frameworks as regards medical plants to embrace more experimentally ascertained facts. This was also a period of artistic flourishment, when the printing press was invented which facilitated the dissemination of literary works [22]. Incidentally, one of the first *incunabula* (i.e., earliest printed books, between 1450 and 1500), the famous and obscure *Hypnerotomachia Poliphili* (Poliphilo’s Strife of Love in a Dream) attributed to the Italian Dominican priest Francesco Colonna (c. 1433–1527), features several mint species among the numerous botanical varieties described in the story’s oneiric landscapes [23]. The 16th century was marked by the rupture with the orthodox medical doctrine based on the theory of humors, and a new doctrine emerged called *nova medicina*, which was rooted in alchemy, astrology, magic, and natural philosophy. The advent of iatrochemistry made possible for new plant extracts and essential oils to be prepared, some from the recently discovered New World, which increased the number and variety of available medicine products [24]. The development of pharmaceutical technology from the 19th century onward has largely contributed to the diversification of mint-based products [25]. Peppermint essential oil, for example, became such a popular medicine in the 19th century that it found its way into the post-impressionistic painting *Still Life with Peppermint Bottle* by French painter Paul Cézanne [26]. Apart from being a source of inspiration to artistic expression, mints also aroused curiosity in the emerging scientific fields. For example, one mint species was used by Joseph Priestley (1733–1804) in his pioneering studies on photosynthesis [27], while Charles Darwin (1809–1882) used peppermint essential oil for his studies on botanics [28]. Mint active compounds concentrate mainly in the leaves of herbs, which can be used by dry grounding them into powder or fresh and being subjected to solvent extraction or steam distillation, the latter of which produces mint essential oils. These oils contain a variety of volatile compounds and are widely used in food, cosmetic, and perfume industries, largely for their flavoring, fragrance, and preservative properties, which make them very economically valuable. The cultivation and processing of mint herbs is a very large business and one that is responsible for a considerable part of the economy of many countries [4].

Most of the data regarding the medical and scientific uses given to mints throughout history are vastly scattered and/or incomplete. The aim of this paper was to provide an extensive descriptive overview of the medical uses given to these herbs, highlighting both the authors in medical culture responsible for their dissemination, as well as their major galenic formulations. The next section references these authors and respective written sources in chronological order (Table 1), together with a concise and up-to-date appreciation of these medical uses in light of ongoing scientific research (Table 2). A comprehensive review of the composition of the mint herbs and a correlation with their ancient/actual medical properties is beyond the scope of this paper. Databases on medical science (Pubmed, Springer Link, Google Scholar, Internet Archive, U.S. National Library of Medicine) were searched using combinations of the following keywords: “mentha”, “mint”, and its variations to include the several species’ names, “medical” and “history”. Reference textbooks on medical history, botanics (aromatic herbs), and pharmacognosy were also consulted. The literary works of the most relevant authors were accessed and analyzed as regarding the previous keywords. From the numerous medical/science research papers accessed, the most relevant ones for the historical discussion comprised in this paper were selected. Given that some authors of the many analyzed written sources do not clearly identify the mint species used, the term “mint” will be used throughout this paper with the meaning “unknown species”. Nevertheless, whenever possible, the names of the concerned species will be provided.

## 2. Medical Applications

### 2.1. For Scenting and Perfuming

Probably the most recognizable quality of mints is their appealing fragrant scent, which is why they have been used since early times in embalming funerary rites. The embalming practice continued until the Middle Ages in Christian Europe but was reserved to the elite. In fact, mint was recently found among the embalmed remains of King Richard the Lionheart (1157–1199) [29] and of John Plantagenet of Lancaster, first Duke of Bedford (1389–1435) [30]. The purpose of using aromatic herbs, such as mints, in these practices was not only to create conditions for long-term body conservation but to also confer it a good odor, similarly to the body of Christ (i.e., the sanctity odor) [29].

Mints were also employed to manufacture perfumes and to mask distasteful substances in medicines. For hygienic purposes, Paulus Aegineta (625–690), a Byzantine Greek physician and compiler of Greek–Roman medicine, best known for his encyclopedia *Medical Compendium*, mentioned the use of ground pennyroyal in a mint decoction, among several ingredients, to mask the displeasing taste of medicinal draughts [31]. Peppermint essential oil is featured in several formulations containing either laudanum (i.e., an opium tincture) or morphine (i.e., the main component of opium). Albert Ethelbert Ebert (1840–1906), a prominent pharmacist of the late 19th century, featured peppermint essence in *The Standard Formulary* as an ingredient of a complex formulation in which morphine was included [32]. Similar uses were later carried out to mask the odor of fish oil [33], cod liver oil [34,35], and laudanum. An example of the last case was the inclusion in the infamous nostrum *Shiloh’s consumption cure* to be used for colds, coughs, bronchitis, asthma, and irritation of the throat [36]. Unquestionably, the most explored mint compound for scenting and perfuming is menthol. This is easily explained by its capacity to induce the perception of coolness, which can strongly affect cognition, emotion and behavior [37]. It is legitimate to interpret that, given the inflammatory component of many diseases, manifested topically with redness and warmth, a “cooling” or “fresh” sensation would be logically sought for to calm the effect. Furthermore, the known local menthol anesthetic seems to have been also an important reason to include these herbs in medical recipes.

### 2.2. For Gastrointestinal Disorders

The medical use of mints for gastrointestinal affections is present in the works of most philosophers and physicians who came across them, from classical antiquity to present-day medicine. Mints have been consistently referred to as possessing anti-emetic and carminative properties, and being used to facilitate digestion and assist in the treatment of gastrointestinal disorders. Pliny was among the first to document these properties and, quoting Democritus, wrote that mints were a suitable treatment for vomiting [17]. Aëtios of Amida (502–575), a Byzantine physician and writer, credits Kyrillos, the archbishop, with a recipe of a digestive composed of a mixture of plants, including pennyroyal macerated in vinegar [38]. Paulus Aegineta left many references to the digestive benefits of mints. For stomach problems, he advised drinking ‘a draught of juice of endive sprinkled with mint’ or ‘a mixture of juice of kernel, pomegranate and mint’ [31]. Centuries later, Hildegard von Bingen (1098–1179), a German Benedictine abbess and polymath, left many texts about the use of medical plants in her *Physica*. To adding to her theoretical knowledge, she gained practical experience in the use of the plants that she grew in her monastery’s garden. To ease digestion, she advised consuming watermint and spearmint for they ‘warm the stomach’ [39]. Trota, a prominent figure of the medical school of Salerno in the 12th century, was another female healer and medical writer to have contributed to extend knowledge on female healthcare. In her *Trotula*, a famous compendium of texts on women’s medicine, she detailed several recipes involving mints [40]. For constipation one recipe consisted on cooking mint in honey and water, a beverage that was to be drunk by after bloodletting. Another recipe consisted in a mixture of wild celery, mints, cowbane, mastic, cloves, watercress, madder root, sugar, castoreum, zedoary, and gladden. This mixture was to be made into a very fine powder and be given with wine to relieve abdominal distension caused by trapped gas during pregnancy and the consequent risk of miscarriage. Another mention in the *Trotula, Potio Sancti Pauli* (Saint Paul’s Potion), a potion including horsemint, was made to diverse gastric ailments. With the extended use of mints throughout Europe, several recipes began to appear among the royal apothecaries’ arsenals. For example, a recipe for a plaster made of wheat bread, cumin, wormwood, mint, and rose leaves figures in the long list of medicines to Katherine Neville, Duchess of Norfolk by John Clerk (15th century), king’s apothecary to Edward IV, which represents both the fear of epidemics and the need to correct the excesses of the aristocratic lifestyle [41]. Mint medicines also appear in the apothecary’s list for Anne of Bohemia, first wife of King Richard II. Although the use given to mints in these lists is not specified, the intended treatment of gastrointestinal disorders is probable [42].

Theophrastus von Hohenheim (Paracelsus, 1493–1541), the German-Swiss physician and alchemist, is credited with bridging medical practice and chemistry fundaments. For difficult digestions, Paracelsus suggested consuming a mixture of mint water and syrup of gillyglower [43]. Garcia de Orta (1501–1568), a Portuguese physician and herbalist who pioneered tropical medicine while working in the empire’s eastern colonies, wrote an important medical treatise on pharmacognosy entitled *Colóquios dos simples e drogas da India* (Conversations on the simples, drugs and medicinal substances of India). In this work, Garcia de Orta advised mixing mint water and mastic powder for ‘vomiting and weakness of the stomach’ [44], which would be later referenced by the also celebrated Portuguese physician and naturalist Cristóvão da Costa (1515–1594) in his *Tractado de las drogas y medicinas de la Indias Orientales* (Treatise of the drugs and medicines of the East Indies), based on Garcia de Orta’s own work [45]. Prosper Alpinus (1553–1617), a Venetian physician and botanist, referenced a treatment for bile vomiting consisting in the administration of ‘sub-acid fruits, juice of wormwood or of mint in wine’ [31]. Herman Boerhaave (1668–1738), the prominent Dutch physician, was also interested in mints for their usefulness in digestive ailments. In his *Materia medica* (On Medical Material), he wrote about watermint and peppermint as anti-emetics [46]. Thomas Sydenham (1624–1689), the prominent English physician, used mint water as a solvent for several anti-emetic agents [47].

The carminative (i.e., reliever of flatulence) and spasmolytic properties of mints were also much appreciated. Paulus Aegineta provided a recipe for constipation in children, for which he advised rubbing the abdomen with a mixture of mint and honey [31]. Robert Burton (Democritus Junior, 1577–1640), Oxford scholar and author of the celebrated *The anatomy of melancholy*, described watermint as an effective carminative [48]. The prominent English physician and founding member of the Royal Society, Thomas Willis (1621–1675), wrote recipes using mint as an anti-emetic and antispasmodic in his *Dr. Willis’s Receipts for the Cure of All Distempers* [49], while Sydenham used watermint to relieve the so-called ‘iliac passion’ (i.e., intestinal volvulus) [47]. Reverend Joseph Townsend (1739–1816), English physician and vicar, listed peppermint as an effective spasmolytic in his *Elements of Therapeutics: or, a Guide to Health; being Cautions and Directions in the Treatment of Diseases* [50]. In the late 19th century, a nursing book by Isabel Robb (1860–1910), a nurse theorist, mentions the internal application of peppermint water to ameliorate colic in infants [51].

The gastrointestinal usefulness attributed to mints has been uncovered in recent scientific publications. Mint oils possess substances that increase gastric emptying to improve digestion [52] and relax the bowel [53,54]. Its antiemetic properties are of considerable magnitude as they can reduce postoperative, chemotherapy-induced nausea and vomiting [55,56]. Their spasmolytic properties are known to relieve symptoms of irritable bowel syndrome [57,58,59]. They are safe to be used in endoscopic procedures, as a suitable alternative to conventional spasmolytics, and increase the diagnostic sensitivity of the procedure itself [60].

References to intestinal infections, especially of parasitic etiology, can be found in numerous medical treatises, and mints appear as useful therapeutic herbs. Pliny advised taking dry powdered mint in water to expel intestinal worms [17], a reference which was imported into one of the most read works in the Middle Ages, *Macer Floridus*, a hexametric poem on medicine and botanics, presumably authored by Odo de Magdunensis (1070?–1112?) [61,62]. Dioscorides recognized the ability of spearmint to kill roundworms [63]. The Babylonian Talmud advises eating pennyroyal with seven white dates to kill intestinal worms caused by eating raw meat [64]. Paulus Aegineta advised the external application of ‘mint or gith in rose-oil’ to the navel for worm infection and to also give ‘sebesten plums and mint’ to aid low fevers brought up by worm infections [31]. Alexander of Tralles (525–605), a prominent physician from the early Byzantine period and an apparently accomplished helminthologist, also listed watermint among several effective antihelminths [65]. Centuries later, the *Regimen Sanitatis Salernitanum* (The Salernitan Rule of Health), a medieval didactic poem from the Salerno medical school, briefly refers to mints as antihelmintic herbs [66].

The use of mints to treat cholera is described in several medical texts, with the first being once again attributed to Pliny [17]. For cholera, Paulus Aegineta advised drinking ‘juice of pomegranate sprinkled with mint’ [31]. Sydenham used mint as a component of a nourishing drink for cholera patients [47]. Physician William Currie (1754–1828) who, in the 19th century described the disease in his work *Of the Cholera*, also used mints as therapeutic herbs. He also recommended using peppermint water as a palliative treatment for digestive and spasmodic problems and mentioned the activity of mints’ oils against a multitude of pathogenic microorganisms, including *Vibrio cholera*, the causative agent of cholera [67]. Recent studies have shown that the essential oils of several mint species have shown in vitro activity against *Echinococcus*, *Trichostrongylidae*, and roundworm parasites [68,69,70], as well as in vivo activity against *Giardia* and *Entamoeba* species, to name just a few [71].

### 2.3. For Reproductive Purposes

Mints have had vast reproduction applications for not only female hygiene and contraceptive purposes but also for their abortifacient properties. The first records on female health date back to ancient Greece, where contraception was almost limited to the so-called “barrier methods”. Mints were added to pessaries, especially balls of wool, inserted in the female reproductive tract, probably for the cooling/calming sensation they evoked [31,72]. As for hygienic measures, mints were used for washing female genitalia after coition. Soranus of Ephesus (98–138), a Greek physician best known for his gynecological and obstetric work *Gynecology*, prescribed pennyroyal for sitz baths [73]. Similarly, John of Gaddesden (c. 1280–1361), a famous practitioner in 14th century England and the author of *Rosa Medicinae*, advised using watermint for the hygienic washing of female genitalia [74].

The best-known use of mint herbs for female health undoubtedly comes from their recognized effect of increasing uterine contractile strength (i.e., oxytocic). They were used as emmenagogues, especially in the clinical context of dysmenorrhea. Galen (129–c. 210), in his *On the Mixtures and Powers of Simple Drugs*, suggested using watermint and pennyroyal as complementary treatments to bloodletting, a popular treatment for plethoric ailments, and mentioned that they ‘bring on an abundant menstrual flow’ [75]. Centuries later, Luis de Mercado (1525–1611), physician to Phillip II of Spain, also listed pennyroyal as an herb to relieve menstrual retention [76]. The most popular mint for its abortifacient properties was pennyroyal, which has been featured in numeral medical treatises, as well in mythological texts. In the Homeric *Hymn to Demeter*, there is a reference to a recipe for *kykeon*, a porridge containing honey-sweet wine, barley, water, and pennyroyal that was drunk by the goddess Demeter herself. This reference is probably a connotation between pennyroyal and female health and sexuality, but whether it precedes the medical use of the herb or was responsible for it is still a matter of discussion [77]. Pliny described pennyroyal and other mints as being capable of increasing uterine contractions and to ‘help expel the placenta and a dead fetus’ [17], a belief later repeated by Dioscorides [78]. Quintus Serenus Sammonicus (d. 212), the Roman author of *Liber Medicinalis*, a didactic poem on medicine, advised administering pennyroyal in tepid water to induce abortion in women with pregnancies less than 1 month old and whose ‘embryo was weak’ [79], a practice also mentioned in Odo de Magnudensis’ work [61]. Pennyroyal is referenced in *Acharnians*, *Peace*, and *Lysistrata*, three plays by the celebrated dramaturge Aristophanes (c. 450 BC–c. 388 BC). In *Peace*, pennyroyal is suggested to have contraceptive properties [79], and in *Acharnians* and *Lysistrata*, the blossoming of pennyroyal is mentioned as a metaphor for pubic hair [80,81,82]. Trota advised the anointment of pennyroyal oil on a cloth to be placed in the vagina to induce receding of prolapsed uterus caused by traumatic coitus due to the ‘excessive size of the male member’. Pennyroyal also appears in Trota’s work as an herb to be applied in a bath, among several others, which ‘flegmatic and emaciated’ women who could not conceive should take to increase the chances of fertilization. Trota also described several recipes for stimulating menses. In a simple recipe, honey was to be cooked in mint water; mint also appeared in the herb mixture *Tyriaca diathessaron*. A mixture of mint, pennyroyal, rue, red cabbage, leek, and salt would be cooked together in a plain pot, and be drunk in the bath. In addition, she advised the fumigation of cumin, fennel, dill, calamint, mint, and nettle, either individually or mixed. Furthermore, a mixture of ground castoreum, white pepper, costmary, mint, and wild celery in white or sweet wine to be drunk in the evening also appeared. To help women having difficulties to give birth, mint and wormwood powder would also be given, besides mint and other odoriferous herbs being applied to the cervix during the prepartum period. The beverage *Tyriaca magna Galeni*, taken with mint water, was also given to stimulate menses or parturition [40,83]. Centuries later, Hildegard von Bingen wrote in her *Physica* that eating pennyroyal ‘acts to expel the afterbirth’ that remained inside the uterus after delivering the fetus [39]. In the 17th century, James Primerose (d. 1659), English physician and notable opponent of William Harvey’s Theory of Circulation, who dedicated a large portion of his career to gynecology and obstetrics, had also mentioned pennyroyal to be an abortifacient [76]. Nicholas Culpepper (1616–1654), physician, astrologer, and herbalist warned in his *A Directory for Midwives* ‘give not [pennyroyal] to any that is with Child, lest you turn Murderess’ [84]. The almanacs of two female authors of the 17th century England, Sarah Jinner of London (fl. 1658–1664) and Mary Holden of Sudbury (c. 1648–1726), transmitted classically-based medical cures for women, challenging the existing medical hierarchy [85]. Sarah Jinner, a student of astrology and a contemporary of Primerose, wrote the first series of almanacs that focused on female health and destined to transmit updated gynecological knowledge from the medical elite to common literate persons and rural physicians [86]. These almanacs contained herbal useful remedies for managing and treating gynecological disorders, among which a list of ‘pills to expel a dead child’ is included, several containing pennyroyal as an ingredient, which she also noted as being able to regulate menses [87]. Thus, pennyroyal became a household herb for inducing abortion and is reported to have been taken with gin during menstruation as recently as the 1950s [88]. The strong effect of pennyroyal is attributed to the oxytocic terpene pulegone, which has, nonetheless, an important hepatotoxic profile [89].

Regarding male health and fertility, several considerations regarding the possible effect of mints on sperm generation existed. Pliny wrote that adding watermint to milk prevented it from curdling and thickening or turning sour. Assuming that a similar effect would occur with semen, Pliny wrote that ingesting watermint could change the consistency of semen and, therefore, affect fertility [17]. Dioscorides also believed that mints, if taken in large quantities, changed sperm quality and affected erection [31]. Aëtius of Antioch (d. 367), Syrian bishop and physician, wrote that watermint consumption ‘generates much semen, but of a feeble nature’ [31]. Avicenna wrote about watermint as a spermicide, used as a female suppository before coition [74]. In contrast, Trota wrote that men should apply pennyroyal (presumably externally), among others, to increase fertility [40]. Thus, pennyroyal was a valued herb through the ages for female health, but, in the 20th century, its use has steadily declined mainly due to the creation of abortifacient drugs and to the increase in the awareness against this herb’s toxicity profile, with only scarce records of its use being found today.

### 2.4. For Modulating Libido

Reports of mints’ effects on libido are diverse and often controversial. This may be partly explained by the differences in used species and also in the quality and quantity consumed. An obvious consensus was reached by classical philosophers that watermint was aphrodisiac. This notion was shared by Aristotle, Dioscorides, Galen, and, centuries later, by the Persian polymath Muhammad ibn Zakariya al-Razi (Rhases, 854–925) [31] and Nicholas Culpeper in his *The English Physician Enlarged* [90]. Contrary records exist but, strangely enough, appeared only in the Middle Ages. Avicenna recommended taking watermint as a treatment to reduce sexual desire [91], and a similar description is present in Hildegard von Bingen’s *Physica* [39]. Similarly, Trota wrote that mints could be given to placate the repressed sexual desire [40]. Later, the Italian physician Paolo Zacchia (1584–1659), considered one of the fathers of forensic medicine, believed, in accordance with Hippocrates’ beliefs, that eating watermint was responsible for erectile dysfunction [92]. However, the mints’ effect on libido have generated only a modest interest thereafter. Recent animal and clinical studies agree that ingestion of mints have an anti-androgenic effect in males, which might affect erection and may also decrease libido [93].

### 2.5. For Repelling Insects and for Animal Bites

Providing protection against the different elements of the natural world, including animals, was always a concern for human beings, and mints have been employed as insect repellants and adjuncts in animal bite treatments since ancient times. By quoting Xenocrates (396 BC–314 BC), Pliny advised smelling pennyroyal and placing it near patients with tertian fevers (probably referring to malaria) that were prevalent in Hellenistic Greece and Egypt [17]. Trota also mentioned the utility of medicinal drinks *Tyriaca diatesereon* and Saint Paul’s potion for patients with quartan fever (once again, probably malaria) [40]. These practices would likely have been intended to prevent *Plasmodium* protozoa from spreading, the causative agents of malaria from mosquito bites based on herbs’ insect repellant activity. Paulus Aegineta noted that spreading herbs, including pennyroyal, was useful for repelling reptiles [31]. It has already been established that *Mentha* herbs, especially pennyroyal, have repellant, larvicidal and growth/reproduction regulatory activities against a wide variety of insects, including the mosquitoes responsible for spreading malaria (*Anopheles sp.*), yellow fever, dengue (*Aedes aegypti*), and zika (*Culex quinquefasciatus*) [94,95,96,97,98].

Mints were also valued for their scent in the Black Plague pandemic of the Middle Ages, which decimated at least one third of the European population. During this period, a vinegar-based formulation against the Black Plague called the “Four Thieves Vinegar” is thought to have originated in Medieval France, created by thieves that plundered the dead and dying plague victims. It is thought that, because they smeared this formulation on their skin, they were able to come in contact with their victims without being themselves affected [99]. Mints do not appear in the original formulation but were added to later variations of this formulation [100,101]. In this time period, it was still believed that (infectious) diseases were spread by miasmas, disease carrying vapors that emanated from corpses and decaying matter or from the breath of infected persons, a theory introduced by Hippocrates and Galen [102]. The discovery that plague was transmitted by fleas carrying the causative microbe *Yersinia pestis* was made centuries later. For these reasons, it is more logical that mints were added for their ability to offset pestilent odors rather than for their flea-repellant property; in fact, recent studies suggest that mint oils show only a weak flea repellant activity [103].

For the purpose of dealing with animal bites, several records are noteworthy. Nicander of Colophon used mints to create a ‘repellant stench’ to chase off snakes [5], while Pliny wrote of watermint and pennyroyal’s usefulness for snake, scolopendra, and scorpion bites [17]. Trota also suggests people drinking *Tyriaca diatesereon* who had been bitten by poisonous animals and rabid dogs, as well as applying it to the wound itself. She also advised using *Tyriaca magna Galeni* with added mint water to help with poisoned wounds [40]. It is noteworthy that later references to mints are lacking, and recent scientific publications that have addressed the usefulness of mint-derived products to counteract animal venoms are scarce and inconclusive [104].

### 2.6. For Respiratory Disorders

Mints were also used to control respiratory ailments, although literary sources are not abundant. Plutarch (46–119) in his *Moralia* wrote of the habit of Heraclitus, the Greek philosopher of Ephesus, of drinking cold water with spigs of pennyroyal before giving a speech [105]. Pliny made a similar reference and advised taking watermint juice ‘when a person is about to engage in a contest of eloquence, but only when taken just before’ [17]. For lower respiratory tract problems, Theodorus Priscianus (b. 300), a physician from the 5th century Byzantine empire, used pennyroyal in an herbal mixture for chestpain [38]. Aëtios of Amida wrote of a cough medicine containing pennyroyal in a mixture of pepper, hyssop, terebinth, fresh butter, and honey [38]. Gil Rodrigues de Valadares (Giles of Santarém, 1185–1265), a Portuguese Dominican friar and physician, wrote mint-based medicinal preparations, which can be found in *Códice Eborense CXXI/2-19*. For aphonia, Giles of Santarém prescribed a mixture of mint juice, ground pepper, malva seeds, egg yolk powder, and honey to be applied to the tongue [106]. Another interesting reference found centuries later is made to Jean-Paul Marat (1743–1793), the famous French physician, scientist, and politician from the French Revolution. Marat prescribed a mixture of mint and tolu balms and vegetable extracts, as well as a secret nostrum, to treat the Marquise de Laubespine from tuberculosis, a case that increased his popularity among French elites [107]. Research has shown that mints’ compounds create the perception of nasal decongestion but also display bronchodilator and antitussive properties [108,109,110]. Indeed, the menthol-based Vicks^®^ chestrub is extremely popular in the United States for treating common colds [112].

### 2.7. For Cardiovascular and Urinary Disorders

There are only a few references made to using mint herbs for cardiovascular and urinary systems. Pliny wrote of pennyroyal as a diuretic and as one capable of removing bladder stones [17], an idea also defended by Aulus Cornelius Celsus (Celsus, c. 25 BC–c. 50) in regard to spearmint [112]. Later, Trota also advised using horsemint and pennyroyal in baths or fumigations to help with strangury (i.e., slow and painful discharge of small volumes of urine), likely due to their diuretic effect [38]. Interestingly, recent studies have shown that menthol does indeed have beneficial effects on the urinary system.

For example, menthol administration is associated with an improved inflammatory profile and clinical manifestations in female patients with interstitial cystitis [113]. As for their cardiac effect, Avicenna wrote of watermint in his opus *Canon of Medicine* as a valuable herb for heart conditions, especially palpitations [114]. Recent research suggests that the *Mentha x villosa* species has a hypotensive effect through bradycardia and vasodilation [115]. Although several mint substances may display this vasoactive effect, it has definitely been detected in menthol [116].

### 2.8. For Pain and Inflammation

Although the pathophysiological mechanisms of inflammation were not uncovered until the 20th century, its central concept was understood many centuries before by Celsus [117]. Accordingly, mints were used either topically or systemically to control the inflammatory manifestations of several diseases, including pain, erythema, and fever, since classical antiquity. Pliny used mints in formulas for cases of ophthalmic and oral inflammation [17]. The Talmud mentions using pennyroyal in a mixture of boiled herbs to be applied to the scalp to ‘soften the skull’ prior to cranial surgery, which would suggest that pennyroyal may have been used as a local anesthetic in ancient Jewish medical practice [118]. Giles of Santarém advised applying a complex mixture of vegetable products, including mint juice, for abscess pain and otalgia. One of these preparations was used as a rubefacient to be applied to persons with leg atrophy, probably as a result of poliomyelitis, demyelinating diseases or even trauma [106]. Thomas Sydenham used liniments containing multiple herbs, including mints, for local applications to the abdomen and armpits in patients with ‘scrophular diseases’, which probably refer to cervical tuberculous lymphadenitis and rickets [47].

Odo de Magdunensis mentions a mixture of mint, strong rue, tansy, and milk cooked in olive oil with virgin wax, made into a plaster and applied to the kidney area of women who could not deliver their child. This could be to relieve pain [83]. Still on the subject of ophthalmic afflictions, William Mackenzie (1791–1868), author of the *Practical Treatise of the Diseases of the Eye*, one of the first British textbooks of ophthalmology, used a formulation of peppermint water, camphorated spirits of opium tincture and borax as a local anesthetic to be applied to the lacrimal puncta prior to mechanical unblocking with a hair pencil [119].

Gout is an inflammatory arthropathy characterized by the painful swelling of joints resulting from the precipitation of uric acid crystals. Mints are among the several types of treatment tried for this disease throughout history. Pliny exalted the usefulness of pennyroyal for treating gout, presumably by covering the affected body region with the herb [17]. In the work of Hildegard von Bingen also mentioned the usefulness of mints for treating gout [39]. Sydenham, having himself been afflicted with gout in the last years of his life, made very accurate descriptions of the disease and included mints in several electuaries (i.e., medicines, generally in powder, mixed in with a palatable medium) form to be taken by patients, as well as for rheumatism [47]. The anti-gout effect of mints may have a scientific basis as recent studies have shown that mint extracts inhibit xanthine oxidase *in vitro*, an enzyme involved in the formation of uric acid, as well as in generating oxidative stress that contributes to the pathophysiology of the disease [120]. The local anesthetic effect of mints is attributed mainly to volatile monoterpene menthol which, at low concentrations, activates receptors on cold nervous fibers by creating a perception of coolness, whereas it is irritating at high concentrations [121,122].

### 2.9. For Oral Health

The first mention of mint herbs for oral health dates back to a 4th century Egyptian papyrus, probably written by a Christian monk, on which a recipe for toothpaste appeared. This toothpaste was a mixture of ‘one drachma of rock salt, two drachmas of mint, one drachma of dried iris flower and 20 grains of pepper’ [123]. Abu Al Qasim Al Zahrawi (Albucasis, 936–1013) recommended washing or gargling with mint decoctions to help with the ‘swelling and erosion of the mouth, tongue and throat’ brought up by the toxic effects of topically applied mercury, a metal already used in Islamic medicine for its therapeutic value [124]. He also advised inhaling the vapors of pennyroyal fumigation in absinthe and vinegar for a swollen uvula [125]. He added mint to borax for oral hygiene purpose [126]. Nikolaos Myrepsos (fl. 13th century), a physician at the court of John III Doukas Vatatzes at Nicaea wrote in his *Dynameron*, one of the richest treatises of the late Byzantine era, about the virtues of mints for the inflammation of teeth, mouth, and palate [127].

Gilbertus Anglicus (Gilbert of England, 1180–1250) wrote *Compendium Medicinae* (Compendium of Medicine), an important medical treatise of the 13th century, which mentions mints as promoters of oral health. For halitosis caused by teeth or gum decay, Gilbertus advised a mouthwash made from birch and mint soaked in wine, after which a linen cloth would be rubbed against gums until they hemorrhaged. Finally, the patient should chew marjoram, oregano, mint, and pellitory leaves, as well as rub the mixture into gums [128]. Paulus Aegineta wrote that ‘Pseudo-Dioscorides recommends mint triturated with honey, red sumach, and rose oil with honey, or by itself for cleaning the tongue’ [31]. Guy de Chauliac (1300–1368), the famous surgeon and pioneer in odontology, advised in his magnum opus *Chirurgia Magna* (Great Surgery) using wine and mint mouthwashes for discolored teeth. For carious teeth, Chauliac advocated an antiseptic gargle made of wine mixed with mint, sage, and pepper or pellitory [129]. For odontalgia, Rev. Townsend references one of Boerhaave’s own prescriptions for a pill to be applied to the decaying tooth composed of opium, camphor, oil of cloves, and peppermint essential oil [50]. Despite the many recipes found for oral health that included mints, obviously the primary therapeutic intention was to create a local anesthetic/anti-inflammatory effect on the affected site.

### 2.10. For Cutaneous Disorders

A few references are made about using these herbs for dermatological purposes. One famous effect, apparently to have been discovered by chance, refers to the treatment of elephantiasis. According to Pliny, elephantiasis was prevalent while Gnaeus Pompeius Magnus (Pompey the Great, 106 BC–48 BC) governed and had been imported from Egypt. He wrote that a person, ashamed of being affected with a facial form of the disease, smeared watermint on his face with shame and was cured [17]. A similar account comes from Paulus Aegineta, who mentions a mixture of ‘watermint, juniper and mezereon’, previously used by Marcellus Empiricus (fl. 385) and Quintus Serenus Sammonicus (d. 212), both medical writers from the late Roman empire [31]. The latter described remedies for different skin problems, such as ‘juice of the bark of the juniper, the ashes and blood of the weasel, mint’ [31]. Paulus Aegineta advised ’washing the head frequently with a lotion made from marjoram, mint or centaury’ for porrago favosa (i.e., favus, a dermatophytosis) [31]. Theophanes Chryssobalantes (fl.c. 950), chief physician of the educated Emperor Constantine VII the Porphirogenitus, in his book *Epitome*, quotes a preparation of the ancient physician Archigenes (1st century AD) that consisted of laudanum and mint in equal quantities for alopecia [130]. Theophanes also indicates a concoction made of celandine (swallow-wort), Egyptian rose, and mint to dye the hair blonde [130].

In *Trotula*, Trota advises anointing *Theriaca magna Galeni* or ‘juice of mint’ to treat the thickness of lips [40]. William Augustus Hardaway (1850–1923) in his *Manual of Skin Diseases* mentions using peppermint essential oil in a formula for ameliorating generalized pruritus in urticaria and papular eczema, which included carbolic acid, glycerin and water, which would then be sprayed onto skin with an atomizer [131]. Recent research shows that several mint-based products appear promising to treat dermatological conditions. Peppermint essential oil is effective for controlling pruritus and for relieving irritation and inflammation [132,133].

### 2.11. For Nervous Disorders

Since ancient times, mint-based preparations have also been known to affect cognition and emotion, and have been used as restorative agents to enable to regain vigor. The oldest record on the effect that mints have on the central nervous system is attributed to Aretaeus of Cappadocia (fl. 2nd century), who advised that epileptics should take walks among acrid and aromatic herbs, such as mint and pennyroyal [134]. Pliny also wrote about the use of watermint to control epileptic seizure, besides it helping with hangovers [17]. For the latter, Marcus Terentius Varro (116 BC–27 BC), a roman scholar and writer, advised hanging garlands of pennyroyal in one’s chambers [17]. In contrast, in the *Hippocratic Corpus* it is written that mint should be avoided by epileptics because of its ‘pungent nature’ [135] There are also several records about using mints to treat headaches. Paulus Aegineta treated throbbing headaches and those resulting from heat exposure with pennyroyal and watermint [33]. Galen wrote about a head compress, previously recorded by Asklepiades, but probably attributed to Nikomedes IV of Bithunia, which contained ‘sulfurwort (hog-fennel), rue, mint and other herbs in rose oil’ [31]. Finally, some attempts have been made to use mints for psychiatric disorders. Galen also wrote about hysteria, and, among his treatments, he administered a mixture of plants, such as mint, hellebore, laudanum, belladonna extract, and valerian [136]. In Trota’s work, *Potio Sancti Pauli*, which included horsemint, was given to treat several nervous states, including ‘epileptics, cataleptics and analeptics’ [40]. Centuries later, Rev. Townsend tried peppermint essential oil, without much success, to treat hysterical fits [50], as did John Quincy (d. 1722), the English apothecary and medical writer, with pennyroyal water [137].

**Table 1:** Probable medical uses given to different *Mentha* species by the main authors discussed in this review throughout history in chronological order.

Author	*Mentha* Species	Probable Medical Use
Nicander of Colophon (197 BC–170 BC)	Unknown	Snake repellant
Marcus Terentius Varro (116 BC–27 BC)	Pennyroyal	Anticonvulsant?
Pliny (23-79)	Pennyroyal	Stimulate parturition Abortifacient Insect repellant Treatment of snake, scolopendra, and scorpion bites Diuretic Local anesthetic/anti-inflammatory
	Watermint	Treatment of snake, scolopendra, and scorpion bites Anticonvulsant?
	Unknown	Anti-emetic Antiparasitic Anti-inflammatory
Celsus (c. 25 BC–c. 50)	Spearmint	Diuretic
Dioscorides (40–90)	Pennyroyal	Stimulate parturition
	Spearmint	Antiparasitic
Soranus of Ephesus (98–138)	Pennyroyal	Female hygiene
Galen (129–c. 210)	Pennyroyal	Stimulate menses
	Watermint	
	Unknown	Local anesthetic/anti-inflammatory Antidepressant/antipsychotic?
Quintus Serenus Sammonicus (d. 212)	Pennyroyal	Abortifacient
Theodorus Priscianus (b. 300)	Pennyroyal	Local anesthetic/bronchodilator/antitussive?
Aëtios of Amida (502–575)	Pennyroyal	Gastrointestinal (to Improve digestion)
		Antitussive/expectorant
Alexander of Tralles (525–605)	Watermint	Antiparasitic
Paulus Aegineta (625–690)	Pennyroyal	Reptile repellant Anti-inflammatory/analgesic
	Watermint	Anti-inflammatory/analgesic
	Unknown	Improve digestion Carminative Antiparasitic Antifungic Flavorant
Abulcassis (936–1013)	Pennyroyal	Local anesthetic/anti-inflammatory
	Unknown	
Theophanes Chryssobalantes (fl.c. 950)	Unknown	Treatment for alopecia
Avicenna (980–1037)	Watermint	Spermicide Reduce libido
Odo de Magdunensis (1070?–1112?)	Unknown	Antiparasitic Local anesthetic
Hildegard von Bingen (1098–1179)	Pennyroyal	Stimulate parturition
	Spearmint	Improve digestion
	Watermint	Improve digestion Reduce libido
	Unknown	Local anesthetic/anti-inflammatory
Gilbertus Anglicus (1180–1250)	Unknown	Local anesthetic/anti-inflammatory
Giles of Santarém (1185–1265)	Unknown	Local anesthetic/anti-inflammatory
Trota (fl. 12th century)	Horsemint	Carminative Insect repellant? Diuretic? Anticonvulsant/antidepressant/neuroleptic?
	Pennyroyal	Induce receding of uterine prolapse Increase fertility Diuretic?
	Unknown	Stimulate menses and parturition Reduce libido Anti-inflammatory?
Nikolaos Myrepsos (fl. 13th century)	Unknown	Anti-inflammatory
John of Gaddesden (1280–1361)	Watermint	Female hygiene
Guy de Chauliac (1300–1368)	Unknown	Oral hygiene
Paracelsus (1493–1541)	Unknown	Improve digestion
Garcia de Orta (1501–1568)	Unknown	Anti-emetic
Cristóvão da Costa (1515–1594)	Unknown	Anti-emetic
Luis de Mercado (1525–1611)	Pennyroyal	Stimulate menses
Prosper Alpinus (1553–1617)	Unknown	Aanti-emetic
Robert Burton (1577–1640)	Watermint	Carminative
James Primerose (d. 1659)	Pennyroyal	Abortifacient
Thomas Willis (1621–1675)	Unknown	Anti-emetic Carminative
Thomas Sydenham (1624–1689)	Watermint	Spasmolytic
	Unknown	Local anesthetic/anti-inflammatory Anti-emetic Antiparasitic
Sarah Jinner (fl. 1658–1664)	Pennyroyal	Abortifacient, stimulate menses
Herman Boerhaave (1668–1738)	Peppermint	Anti-emetic Local anesthetic Antidepressant/antipsychotic?
	Watermint	Anti-emetic
John Quincy (d. 1722)	Pennyroyal	Antidepressant/antipsychotic?
Reverend Joseph Townsend (1739–1816)	Peppermint	Local anesthetic Spasmolytic Antidepressant/antipsychotic?
Jean-Paul Marat (1743–1793)	Unknown	Antitussive/antibiotic?
William Currie (1754–1828)	Peppermint	Improve digestion Spasmolytic Antiparasitic
William Mackenzie (1791–1868)	Peppermint	Local anesthetic
Albert Ethelbert Ebert (1840–1906)	Peppermint	Flavorant
William Augustus Hardaway (1850–1923)	Peppermint	Local anesthetic/anti-inflammatory
Isabel Robb (1860–1910)	Peppermint	Spasmolytic

**Table 2:** Description and main results of relevant studies assessing the health-promoting effects of mint-based products.

Authors	Mint-Based Product	Study Type	Species	Main Biological Effect
Innamori et al. (2017) [52]	Peppermint oil	*In vivo*	Healthy human subjects	Acceleration of gastric emptying
Hills and Aaronson (1991) [53]		*In vivo*	Guinea pigs	Relaxation of taenia coli
Zong et al. (2011) [54]		*In vivo*	Sprague-Dawley rats	Stimulation of bile secretion
Anderson and Gross (2004) [55]		*In vivo*	Human subjects (ambulatory surgery patients)	Reduction of post-operative nausea
Tayarani-Najaran et al. (2013) [56]	Spearmint and peppermint oils	*In vivo*	Human subjects (patients undergoing chemotherapy)	Reduction of chemotherapy-induced nausea and vomiting
Asao et al. (2001) [57]	Peppermint oil	*In vivo*	Human subjects (undergoing colonoscopy)	Reduction of colonic spasm
Maggiore et al. (2012) [68]	Pennyroyal and peppermint oils	*In vitro*	*Equinococcus granulosus* (protoscoleces)	Protoscolicidal effect
Katiki et al. (2011) [69]	Peppermint oil	*In vitro*	*Haemonchus contortus* and *Trichostrogylus* spp.	Anthelminthic activity
Girme et al. (2006) [70]	Peppermint extract	*In vitro*	*Pheritima posthuma*	Anthelminthic activity
Akdogan et al. (2004) [93]	Spearmint and peppermint teas	*In vivo*	Male Wistar rats	Decrease in total plasma testosterone levels Arrest of spermatogenesis
Ansari et al. (2000) [94]	Peppermint oil	*In vitro*	*Aedes aegypti, Anopheles stephensi* and *Culex quinquefasciatus* (IIIrd instar larvae)	Larvicidal activity
		*In vivo*		Repellant activity when applied in human skin
Erler et al. (2006) [95]	Peppermint oil	*In vitro*	Female *Culex pipiens*	Repellant activity
Rocha et al. (2015) [97]	Pennyroyal oil	*In vitro*	*Anopheles atroparvus*, *Anopheles gambiae*, *Anopheles stephensi* and *Aedes aegypti*	Larvicidal activity
Eccles et al. (1990) [108]	Menthol lozenge	*In vivo*	Human subjects	Subjective sensation of nasal decongestion
Sharma et al. (2018) [109]	Wildmint oil	*In vivo*	Guinea pigs	Relaxation of bronchial smooth muscle and suppression of immunological response to ovalbumin
Laude et al. (1994) [110]	Menthol	*In vivo*	Guinea pigs	Antitussive (reduction of cough frequency)
Paul et al. (2010) [111]	Vicks VapoRub	*In vivo*	Human subjects (children with symptoms of upper respiratory tract infections)	Symptomatic relief of nocturnal cough, congestion, and sleep difficulty
Shahid et al. (2018) [113]	Menthol	*In vitro*	RAW 264.7 cell line	Suppression of lipopolysaccharide-stimulated cytokine release
Lahlou et al. (200) [115]	Hairy mint (*Mentha X villosa var. alopecuroides* Hull) oil	*In vivo*	Male Wistar rats (under anesthesia)	Hypotension and bradycardia
Elsaie et al. (2016) [132]	Peppermint oil	*In vivo*	Human subjects with chronic pruritus	Improvement of pruritus

## 3. Conclusions

This paper makes a thorough descriptive review of the many medical uses given to mints throughout history, from ancient civilizations to modern day medicine, beyond previously published material, highlighting both the authors in medical culture responsible for their dissemination, as well as their major galenic formulations. Most medical knowledge came from Ancient Greek and Roman philosophers and medical authors, while later contributions consisted mainly of progressive technological improvements. From the immediately perceived qualities of these herbs, it can be inferred that the primary intention of their topical application was to probably cause local anesthesia and to control irritation and inflammation, being especially used in gastrointestinal tract affections. Their particular scent and flavor also came of use for the purpose of masking the unpleasant taste of many medicinal formulae long before the advent of pharmaceutical technology. The longevity and diversity of the use of mints in medicine are a testament of their importance, receiving still noble place in herbal medicine.
